# Barrier Effects of Cellulosic Fibers with Hybrid Coating Based on Zirconium Metal-Organic Framework

**DOI:** 10.3390/polym14153071

**Published:** 2022-07-29

**Authors:** Qiuyue Wu, Manuel Jose Lis

**Affiliations:** 1 The Institute of Textile Research and Industrial Cooperation of Terrassa, Polytechnic University of Catalonia, 08222 Barcelona, Spain; qiuyueywu@gmail.com; 2 Department of Chemical Engineering, Polytechnic University of Catalonia, 08222 Barcelona, Spain

**Keywords:** metal-organic frameworks, cellulosic fibers, fire barriers, polyethyleneimine, vinyltriethoxysilane

## Abstract

Metal-organic frameworks (MOFs) have great potential for the development of fire barriers for flammable materials. Accordingly, zirconium-based metal-organic framework (Zr-MOF), branched polyethyleneimine (BPEI), and vinyltriethoxysilane (VTES) were deposited to produce composites assembled on cellulosic fibers to investigate their barrier effects. The structure, morphology, and thermal properties of the cellulosic fibers were characterized using FTIR spectroscopy, SEM, and TGA. Compared with the untreated cotton sample, the temperature of the maximum rate of weight loss (T_max_) of C-Zr-MOF/BPEI/VTES increased from 479 to 523.3 °C and the maximum weight loss rate (R_max_) at T_max_ decreased from 37.6 to 17.2 wt%/min. At 800 °C, the pristine cotton was burned out without residues whereas the residual char content of the C-Zr-MOF/BPEI/VTES sample was 7.2355 wt%. From the vertical burning tests, the results suggested that the C-Zr-MOF/BPEI/VTES sample had better barrier effects by reducing the flame-spread speed and generating more protective char layers.

## 1. Introduction

Cotton, a natural cellulosic fiber, is popular in the textile industry with a long history due to its superior characteristics of moisture permeability and comfort-dressing [1]. However, compared with wool, silk, and some synthetic fibers, the flash point and ignition point of cellulosic fibers are lower with a limiting oxygen index (LOI) of 18.4% [2,3,4], which are considered as flammable or combustible materials that can easily cause fire accidents, environmental hazards, personal injury, and property losses [5]. Based on the newest report of the World Fire Statistics by International Association of Fire and Rescue Services (CTIF), an average of 3.7 million fires and 40,800 fire deaths per year were recorded in participating countries by fire services from 1993–2019. It is, therefore, of great research value to improve the barrier effects of cellulosic materials to slow the spread of large burning fires and reduce the possibility of fire risks. To achieve this, it is common to develop fire-retardant surface coatings on cellulose fibers and directly introduce flame retardants (FRs) in the spinning process [6,7].

Over the past several decades, to inhibit the combustion of cotton fabrics, halogenated and formaldehyde-based flame retardants, accounting for a large share of the market, have been widely used [8]. They can quench high-energy radicals through the formation of free radicals, acting cost-effectively and efficiently in the gas phase [9]. However, most halogen FRs are accompanied by corrosive or toxic gases and fumes, which are generated during the combustion process, becoming environmental hazards due to their persistence and bioaccumulation and leading to increasing concerns and threats over human health. In 2006, brominated FRs, such as polybrominated biphenyls (PBBs) and polybrominated biphenyl ether (PBDE), were restricted in Europe as stated in the Restriction of Hazardous Substances (RoHS) Directive [10]. Since then, more halogenated FRs have been controlled or banned worldwide to promote halogen-free manufacturing [11]. Hence, the development and application of novel halogen-free FRs, such as nitrogen-based FRs and silicon-based FRs, has attracted a lot of attention recently [12].

Branched polyethyleneimine (BPEI) is a polymer with repeating units consisting of ethylene diamine groups that has high cationic charge density and water solubility. Strong hydrogen bond interactions are generated between the abundant amines of BPEI and the carboxyl groups of cellulose [13], which has the potential to incorporate other flame-retardant elements for synergistic and intumescent effects.

Silicon-based FRs are also widely known due to their high thermal stability and environmental friendliness [14]. In terms of the mechanism, they can form a siliceous carbon layer when the material is thermally decomposed, which can prevent the burning of materials due to heat insulation and the oxygen barrier. Meanwhile, they reduce the overflow of flammable gas to suppress fire [15]. Among a variety of silicon-containing compounds, vinyltriethoxysilane (VTES), a common silane-coupling agent [16], with both vinyl groups and hydrolytically sensitive ethoxysilyl groups [17], can cross-link cellulosic fiber through the Si–O–Si network [18] and promote the silicon-nitrogen synergy system as well.

Metal-organic frameworks (MOFs) are a new type of porous material with organic/inorganic coordination networks [19]. They are stable at high temperatures of typically above 200 °C, exhibit a high degree of crystallinity, and are characterized by a very high surface area [20]. MOFs are considered to inherently possess superior thermal stability, which is comparable with inorganic FRs. Organic ligands can not only contribute to excellent compatibility but also flame-retardant elements or functional groups, such as nitrogen, phosphorus groups, and aromatic derivatives [21]. UiO-66 (Zr-based framework) has been extensively studied because of its broad applications and outstanding stability, and it was reported to grow on the surface of cotton fibers by facile two-step synthesis [22].

Referring to some previous studies [23,24], organic-inorganic hybrid coatings have been utilized for cotton to improve its thermal properties. A multilayer of sodium phytate and APTES was dispersed uniformly on the surface of cotton with the addition of 33.4 wt% to achieve a fire-retardant performance [25]. Further, 20 BLs CS/MMT composites were assembled onto cotton fabric y layer-by-layer, which increased the char-forming ability of cotton from 2.4 to 8.5 wt% residues as revealed by TGA under a nitrogen atmosphere [26]. However, it is also worth noting the reduction in the significant impacts on the physical properties of pristine cotton substrate due to excessive weight gain after treatment and avoidance of the redundancy of the application procedure. In this work, Zr-MOF, BPEI, and VTES were successively deposited to produce composites assembled onto cellulosic fibers to achieve barrier effects. The structure, morphology, and thermal properties of the cellulosic fibers were characterized using Fourier Transform Infrared (FTIR) spectroscopy, scanning electron microscopy (SEM), and thermogravimetric analysis (TGA). The flammability and fire-resistant properties of the cellulose species were evaluated in fire tests. Using the facile and green approach, the findings exhibited that the fire performances of the cotton samples were noticeably enhanced after incorporating the hybrid coating consisting of Zr-MOF, BPEI, and VTES.

## 2. Experimental Section

### 2.1. Reagent and Materials

Commercial cotton twill fabric, 250 g/m^2^, was used for the functionalization treatments. Vinyltriethoxysilane (VTES) was provided by Qingdao Hengda Zhongcheng Technology Co., Ltd. (Qingdao, China). Zirconium chloride, trimesic acid (H_3_BTC), and branched polyethyleneimine (Mw 25,000) were purchased from Aldrich Chemicals company (St. Louis, MI, USA).

Deionized water (DI) was employed in all experiments.

### 2.2. Characterization Methods

#### 2.2.1. Particle Size and Stability of Zr-MOF Homogeneous Dispersion

The particle size of the Zr-MOF homogeneous dispersion was analyzed by a Malvern Zetasizer Nano ZS90 (Worcestershire, UK) using the dynamic light scattering (DLS) technique. The visible stability of the Zr-MOF dispersion was observed in digital photos of the samples at different times.

#### 2.2.2. Fourier Transform Infrared (FTIR) Spectroscopy

The Fourier Transform Infrared (FTIR) spectra of all the samples were measured using a Nicolet iS10 FTIR Spectrometer (Thermo Fisher, Waltham, MA, USA) to identify the functional groups after completion. The scanning wavelength range was 4000–500 cm^−1^ and the spectral resolution was 4 cm^−1^.

#### 2.2.3. Scanning Electron Microscopy (SEM)

The morphology of the cellulosic fibers and the residual carbon were observed using the scanning electron microscope (JSM-5610, JEOL, Tokyo, Japan) installed at the imaging platform center of Polytechnic University of Catalonia (Barcelona, Spain) at an acceleration voltage of 10 kV.

#### 2.2.4. Thermogravimetric Analysis (TGA)

The thermal properties of cellulosic fibers were evaluated using a Thermal Analysis System TGA 2 STARe (Mettler-Toledo, Kuesnacht, Switzerland). All the samples were loaded into an alumina holder and heated from 30 to 800 °C with a heating rate of 10 °C∙min^−1^ and gas flow of 50 mL∙min^−1^. The tests were measured under an air atmosphere. Thermogravimetric analysis (TGA) curves and corresponding data were obtained.

#### 2.2.5. Fire Test

Drawing on the ASTM D6413 testing method, the control and treated fabric strips were investigated on a self-made flame testing setup. Samples measuring 280 mm × 75 mm were fixed at the same position and then exposed to a vertical flame. Subsequently, the burning behavior of all the samples was observed and recorded to evaluate their flammability and fire resistance.

### 2.3. Hydrothermal Synthesis of Zr-MOF Homogeneous Dispersion

The protocol for hydrothermal synthesis of Zr-MOF was inspired by Jia et al. [27]. The Zr-MOF homogeneous dispersion was prepared as follows: Firstly, ZrCl_4_ (2.4 g) was completely dissolved in DI (50 mL) while H_3_BTC (2.2 g) was dissolved in DI (50 mL); with the help of a Turrax Digital Homogenizer, the two were mixed and refluxed in a sealed reactor at 100 °C for 16 h; then, 170 mL of ethanol and water mixture (1:1) was added and followed by ultrasonication treatment for 10 min; after overnight magnetic stirring at room temperature, the homogeneous dispersion of Zr-MOF was achieved.

### 2.4. Finishing of Cotton Substrates with Zr-MOF, BPEI, and VTES

**Figure 1:**
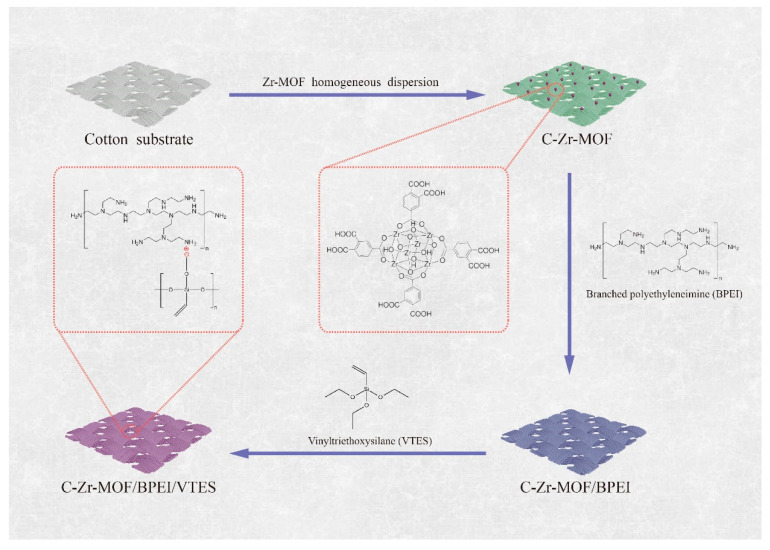
Graphical representation of the hybrid coating assembled onto cellulosic fibers.

VTES solution was prepared by directly dispersing VTES (8 g) into DI (92 mL) by ultrasonication for 10 min and magnetic stirring for 8 h at room temperature. The cotton fabrics were treated by the resulting Zr-MOF homogeneous dispersion first followed by BPEI (3 wt%) and VTES (8 wt%) solution via the dip-pad-dry process as Figure 1 illustrates. The add-on (wt%) of each formulation was calculated from the following equation:

(1) $$\mathrm{Add}-\mathrm{on}\;\left(\mathrm{wt}\%\right)=\frac{\mathrm{Last}\;\mathrm{weight}\;\mathrm{of}\;\mathrm{specimen}\left(g\right)-\mathrm{Initial}\;\mathrm{weight}\;\mathrm{of}\;\mathrm{specimen}\left(g\right)}{\mathrm{Initial}\;\mathrm{weight}\;\mathrm{of}\;\mathrm{specimen}\left(g\right)}\times 100$$

After the finishing treatment, samples were denoted as C-Zr-MOF, C-Zr-MOF/BPEI, and C-Zr-MOF/BPEI/VTES, respectively, with the gained add-on of 2.9, 5.6, and 10.9 wt%.

## 3. Results and Discussion

### 3.1. Particle Size and Stability of Zr-MOF Homogeneous Dispersion

**Figure 2:**
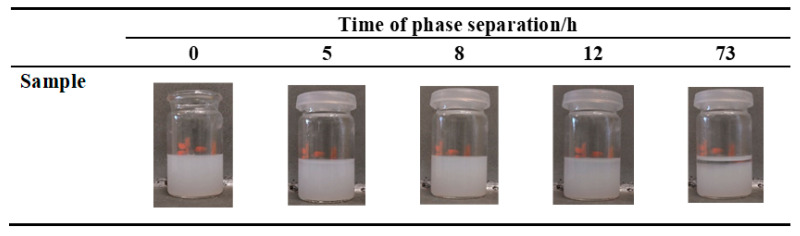
Digital photos of the Zr-MOF homogeneous dispersion at different times.

After successfully obtaining the synthesized Zr-MOF dispersion, the particle size was immediately detected as 381 nm using a Malvern Zetasizer Nano ZS90 (Worcestershire, UK). With such a fine particle size, it is beneficial to distribute Zr-MOF more uniformly on cellulosic fibers and improve the add-on. The evolution of the Zr-MOF dispersion stability with time could be intuitively seen from the digital photos (Figure 2). The gravitational phase separation of the Zr-MOF dispersion gradually appeared after 12 h or more. It is quite practical to maintain a uniform dispersion of Zr-MOF over several hours in ambient conditions.

### 3.2. Fire Test

To evaluate the flammability and fire resistance of the cotton samples, vertical burning tests were conducted. Given the high flammability of cellulosic fibers, these samples were exposed to a naked flame for 10 s before removing the ignition source in order to record the combustion process.

**Figure 3:**
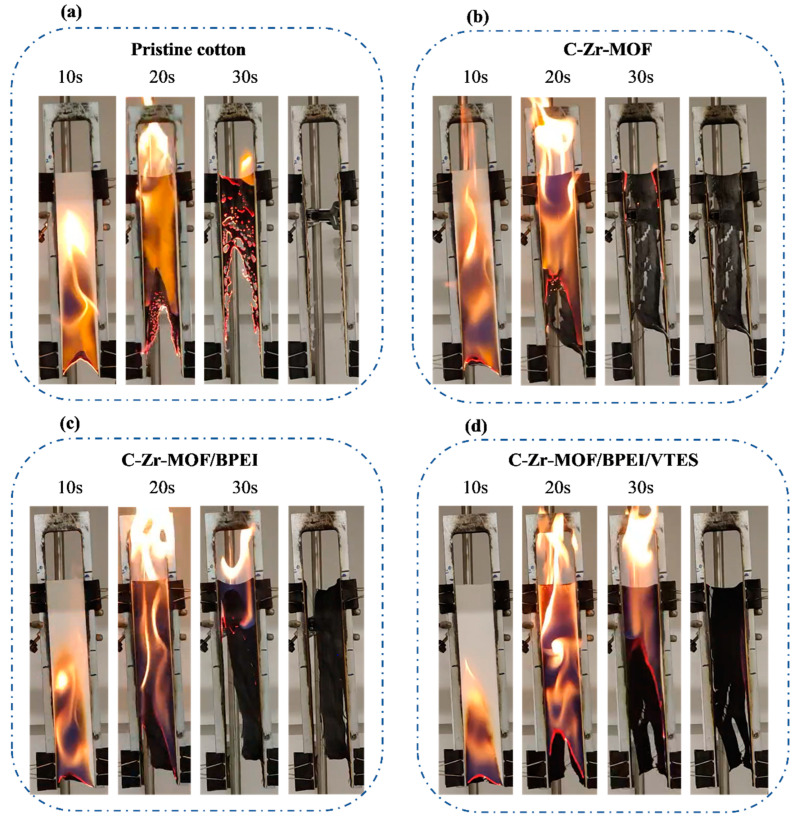
Digital photos of (**a**) pristine cotton; (**b**) C-Zr-MOF; (**c**) C-Zr-MOF/BPEI; and (**d**) C-Zr-MOF/BPEI/VTES in the vertical burning test after ignition.

As seen in Figure 3, the pristine cotton vigorously burnt, and the flame spread extremely rapidly after ignition. At the end of the combustion, the fabric was entirely consumed, without any remaining chars. With the very low add-on (2.9 wt%) of the C-Zr-MOF sample, the combustion duration was shortened accompanied by the formation of a small amount of chars. Additionally, because BPEI acted as an effective carbon source, denser char layers were found for the C-Zr-MOF/BPEI sample. Apparently, neither C-Zr-MOF nor C-Zr-MOF/BPEI appeared to slow the flame spread, suggesting that another flame-retardant element was expected to be introduced to establish the synergetic system. From the analysis of the burning behavior of the C-Zr-MOF/BPEI/VTES sample, it showed better barrier effects by reducing the flame-spread speed and generating more protective char layers at the same time.

### 3.3. SEM

**Figure 4:**
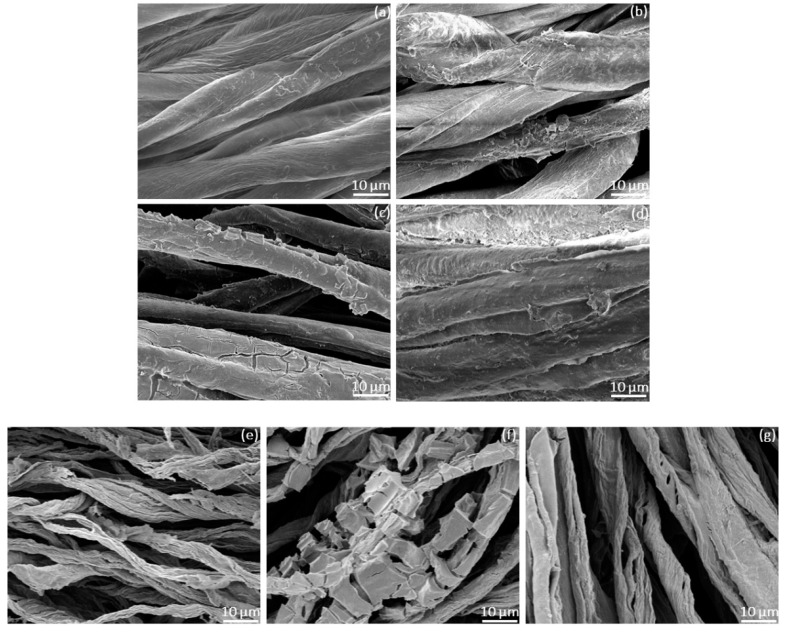
The SEM images of (**a**) pristine cotton; (**b**) C-Zr-MOF; (**c**) C-Zr-MOF/BPEI; (**d**) C-Zr-MOF/BPEI/VTES; (**e**) C-Zr-MOF residual chars; (**f**) C-Zr-MOF/BPEI residual chars; and (**g**) C-Zr-MOF/BPEI/VTES residual chars.

To study the surface morphology of cellulosic fibers before and after burning, all samples were observed using SEM. The morphology of pristine cotton, C-Zr-MOF, C-Zr-MOF/BPEI, and C-Zr-MOF/BPEI/VTES before burning is exhibited in Figure 4a–d.

It can be seen that the surface of the untreated cellulosic fibers was relatively smooth, flat, and clean (Figure 4a). For treated samples, Zr-MOF was adsorbed to form a micron-level rough structure on the fiber surface while Zr-MOF was partially agglomerated into larger-sized particles that may generate certain fiber defects (Figure 4b). As a hyperbranched polymer, BPEI can help Zr-MOF to penetrate the interstices of cellulosic fibers, making them well distributed and forming a more uniform and continuous film-like structure via the bonding effect against fire (Figure 4c). With VTES, the cellulosic fibers were fully coated because of strong cross-linking through the Si–O–Si network, allowing Zr-MOF and BPEI to adhere and be trapped inside as well. The reliable deposition of shielding layers composed of Zr-MOF, BPEI, and VTES provides prospective applications for cellulosic materials, such as fire barrier effects. During the combustion process, the VTES coating could efficiently accelerate the carbonization of cellulosic fibers, considerably increasing the char yield (Figure 4d). After burning, the produced carbonized residuals of the treated samples retained the original cellulosic fiber structure to varying degrees as shown in Figure 4e–g. Particularly, for the C-Zr-MOF/BPEI/VTES sample, the coated organic–inorganic composite contributed to denser and thicker barrier layers to protect the fibers during combustion (Figure 4g).

### 3.4. FTIR

**Figure 5:**
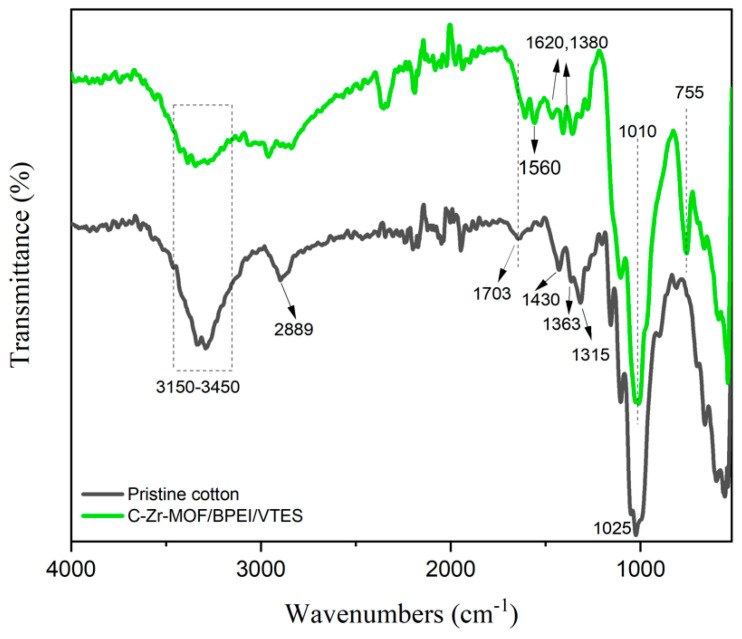
The FTIR spectra of the pristine cotton and C−Zr−MOF/BPEI/VTES sample.

The infrared spectra of the pristine cotton and C-Zr-MOF/BPEI/VTES samples were measured using an FT-IR spectrometer as presented in Figure 5. For untreated cotton, the peaks at 1025, 1315, 1363, 1430, and 2889 cm^−1^ were, respectively, related to (CO) and (OH) stretching of the polysaccharide, C-O bending, C-H bending, CH_2_ bending, and C-H stretching vibration [28]. The broad band at approximately 3300 cm^−1^ of pristine cotton was assigned to the –OH group and weakened after treatment due to the hybrid coating [29,30]. Additionally, the peak near 1703 cm^−1^ representing the free carboxylate group was eliminated in the C-Zr-MOF/BPEI/VTES sample, which demonstrated the formation of a cross-link between the cellulose and Zr-based MOF composites. Owing to the presence of organic linkers, Zr-MOF was highly compatible with cellulosic materials [31]. The positively charged BPEI had a strong electrostatic effect with the carboxyl groups of the cellulose substrate and Zr-MOF. Additionally, VTES was able to interact with both BPEI and cellulose because of hydrogen bonding and van der Waals forces. Some new characteristic peaks could be noted in the C-Zr-MOF/BPEI/VTES sample compared with pristine cotton. The asymmetric stretching vibrations at 1620 cm^−1^ and symmetric stretching vibrations at 1380 cm^−1^ were assigned to the -COO-Zr group [32], confirming the deposition of Zr-MOF on the surface of cotton substrates. While the absorption peaks at 1560 and 755 cm^−1^ were due to N-H bending and N-H wagging from the available BPEI [33], the prominent absorption bands located at 1010 cm^−1^ were identified as the presence of Si-O-Si [34]. The emerging peaks indicated Zr-MOF, BPEI, and VTES were successfully assembled onto cellulosic fibers.

### 3.5. Thermal Properties

**Figure 6:**
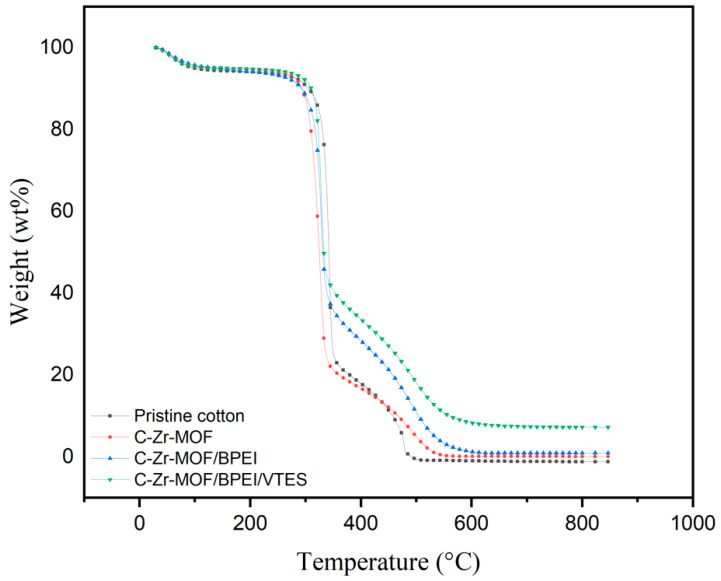
TGA curves of pristine cotton and treated samples.

Due to the hydrophilic nature of cellulosic fibers, the weight loss around 100 °C was related to moisture evaporation [35]. The thermal decomposition process of each sample was composed of two main stages (Figure 6). The first stage was the main thermal decomposition stage, accounting for a higher ratio of total weight loss. For pristine cotton, the dehydration of cellulosic fibers was facilitated, and char formation was increased, ranging from 318 to 380 °C in the first stage [36], and the chars were further oxidized to produce more gaseous combustible products at higher temperatures in the second stage [37]. As evidenced by the TGA data in Table 1, the initial decomposition temperature (T_10_) of both C-Zr-MOF and C-Zr-MOF/BPEI was slightly lower than that of pristine cotton, which was caused by the evaporation of liquid and early decomposition of Zr-MOF or BPEI. In stage 1, T_max_ increased from 479 to 500.9 °C with the aid of Zr-MOF. R_max_ of pristine cotton in stage 1 was 37.6 wt%/min but R_max_ of C-Zr-MOF was 40.8 wt%/min. Similarly, in stage 2, R_max_ of C-Zr-MOF did not change substantially compared to pristine cotton. As a result, the introduction of individual Zr-MOF did not greatly increase the thermal stability of the cellulosic fibers at elevated temperature. BPEI is regarded as a blowing agent, releasing low-molecular-weight molecules such as NH_3_ and causing the carbonaceous residue to become porous [38]. Compared to the utilization of Zr-MOF alone, R_max_ of the C-Zr-MOF/BPEI sample were reduced in both stages 1 and 2. With the composites of Zr-MOF, BPEI, and VTES assembled on cellulosic fibers, the formed siliceous carbon layer of VTES was the first barrier that could suppress the fire by oxygen and heat insulation [39]. Meanwhile, R_max_ was further reduced from 37.6 to 17.2 wt%/min in stage 1 and T_max_ increased from 479 to 523.3 °C in stage 2. No residue was left on the pristine cotton at 800 °C, whereas the residual char content of the C-Zr-MOF/BPEI/VTES sample was 7.2355 wt%. Thereby, the synergistic effects between Zr-MOF, BPEI, and VTES were more conducive to inhibiting the thermal degradation of cellulosic fibers and improving their thermal stability at elevated temperatures.

## 4. Conclusions

Nowadays, the development of fire barrier properties for cellulosic fibers remains a challenging work. Additionally, the use of a facile and eco-friendly approach to replace complicated and time-consuming processes has greater potential for industrial application such as food, pharmaceuticals, clothing, wood, and cosmetics [40,41]. In our research, the organic–inorganic composites consisting of Zr-MOF, BPEI, and VTES were successfully assembled onto cellulosic fibers to build barrier effects. In the fire tests, the flame-retardancy behavior and carbon-forming properties of C-Zr-MOF/BPEI/VTES were positively demonstrated. According to the photos of SEM, the produced carbonized residuals of C-Zr-MOF/BPEI/VTES partially retained the original cellulosic fiber structure after burning. During combustion, the maximum weight loss rate (R_max_) of the cellulosic fibers was reduced from 37.6 to 17.2 wt%/min and the temperature of the maximum rate of weight loss (T_max_) was increased from 479 to 523.3 °C compared to the untreated cotton sample. At 800 °C, the pristine cotton burned without residues whereas the residual char content of the C-Zr-MOF/BPEI/VTES sample was 7.2355 wt%. For the cellulosic fibers after treatment, the thermal degradation was strongly inhibited and the thermal stability at high temperatures was significantly enhanced due to the synergistic effects of Zr-MOF, BPEI, and VTES.

## Figures and Tables

**Table 1:** TGA data of pristine cotton and treated samples under air atmosphere.

Sample	T_10_% (°C)	Stage 1		Stage 2		Residue at 800 °C (wt%)
		T_max_ (°C)	R_max_ (wt%/min)	T_max_ (°C)	R_max_ (wt%/min)	
Pristine cotton	318.5	341.6	37.6	479	93.4	−1.21
C-Zr-MOF	310.8	325.7	40.8	500.9	92.8	0.0573
C-Zr-MOF/BPEI	303.5	326.2	25.6	499	88.1	0.9052
C-Zr-MOF/BPEI/VTES	319.3	330.8	17.2	523.3	84.6	7.2355

The heating rate was fixed at 10 °C/min. T_10_ is the initial decomposition temperature at which 10% sample weight is lost. T_max_ is the temperature of the maximum rate of weight loss. R_max_ is the weight loss rate at the maximal peak (T_max_).

## Data Availability

Not applicable.
